# Serum IL-38 and the rs7599662 polymorphism: novel biomarkers for pediatric idiopathic nephrotic syndrome

**DOI:** 10.1186/s13052-026-02327-1

**Published:** 2026-08-27

**Authors:** Sherin Khamis Hussein, Maha Hosni Morsi, Omayma O. Abdelaleem, Doaa Ezzat Sayed Ahmed, Doha Elsayed Ahmed Hassanein, Marwa A. Ali, Shaimaa Abdelbadie, Ahmed Mohamed Gamal EL Din Wahba, Asmaa Younis Elsary, Heba Safar, Reham Fares

**Affiliations:** 1 Department of Pediatrics, Faculty of Medicine, Fayoum University, Gamma St., Keman Square, Fayoum, 63514 Egypt; 2 Faculty of Applied Health Sciences Technology, Misr University for Science and Technology (MUST), P.O. BOX 77, Giza, Egypt; 3 Department of Medical Biochemistry and Molecular Biology, Faculty of Medicine, Fayoum University, Gamma St., Keman Square, Fayoum, 63514 Egypt; 4 Department of Medical Biochemistry and Molecular Biology, College of Oral and Dental Surgery, Misr University for Science and Technology, 6 October City, Egypt; 5 Department of Clinical Pathology, Faculty of Medicine, Misr University for Science and Technology, 6 October City, Egypt; 6 Department of Biomedical Sciences, College of Medicine, King Faisal University, Al-Ahsa, 31982 Saudi Arabia; 7 Department of Medical Microbiology and Immunology, Faculty of Medicine, Nahda University, Beni Suef, Egypt; 8 Department of Medical Microbiology and Immunology, Faculty of Medicine, Beni Suef University, Beni Suef, Egypt; 9 Department of Pediatrics, Faculty of Medicine, Nahda University, Beni Suef, Egypt; 10 Department of Pediatrics, Faculty of Medicine, Fayoum University, Fayoum, Egypt; 11 Department of Public Health & Community Medicine, Faculty of Medicine, Fayoum University, Gamma St., Keman Square, Fayoum, 63514 Egypt

**Keywords:** Nephrotic syndrome, Interleukin-38, Biomarker, Disease severity, Polymorphism

## Abstract

**Background:**

Idiopathic nephrotic syndrome (INS) is the most common chronic glomerular disorder in children and is characterized by a variable response to corticosteroid therapy. Interleukin-38 (IL-38), an anti-inflammatory cytokine, and its gene polymorphisms may influence disease susceptibility and treatment outcomes. This study investigated the associations between serum IL-38 levels, the rs7599662 gene polymorphism, and disease presence in pediatric INS patients.

**Methods:**

This case‒control study included 88 pediatric INS patients and 74 age- and sex-matched healthy controls. Patients were categorized as steroid sensitive (50%), steroid dependent (25%), or steroid resistant (25%). Serum IL-38 was measured by ELISA, and rs7599662 (C/T) genotyping was performed via real-time PCR. Statistical analyses included nonparametric tests, chi-square tests, Spearman correlation, and receiver operating characteristic (ROC) curve analysis.

**Results:**

Serum IL-38 levels were significantly lower in patients than in controls (17.9 ± 11.4 vs. 38.8 ± 35.6 ng/L, *p* < 0.001). IL-38 was negatively correlated with age (*r* = -0.28, *p* = 0.01), systolic blood pressure (*r* = -0.28, *p* = 0.01), and urinary protein (*r* = -0.38, *p* = 0.001). ROC analysis revealed that IL-38 had moderate diagnostic performance (AUC = 0.687, *p* < 0.001), with 73.7% sensitivity and 63.8% specificity at a cutoff of 18.773 ng/L. The CT genotype was significantly more common in patients (*p* = 0.003), with a protective association observed for the TT genotype under a recessive model (*p* = 0.002). Serum IL-38 levels did not differ among steroid-sensitive, dependent, or resistant subgroups (*p* = 0.32).

**Conclusion:**

This first report of reduced serum IL-38 and the rs7599662 polymorphism in pediatric INS suggests a role for impaired anti-inflammatory responses in disease pathogenesis. Serum IL-38 shows promise as a diagnostic biomarker and may reflect disease pathophysiology, but does not predict steroid responsiveness. These hypothesis-generating findings require validation in larger, prospective, multicenter studies to establish their clinical utility. The primary value of this work lies in generating testable hypotheses for future confirmatory studies rather than providing definitive mechanistic answers.

**Supplementary Information:**

The online version contains supplementary material available at https://doi.org/10.1186/s13052-026-02327-1.

## Introduction

Idiopathic nephrotic syndrome (INS) is the most common chronic glomerular disorder in children, with an incidence of approximately 15–16.9 per 100 000 cases [1]. The disease is characterized by repeated relapses, often triggered by infections [1].

Nephrotic syndrome (NS) is classically defined as a glomerular condition characterized by heavy proteinuria, hypoalbuminemia, hyperlipidemia, and generalized edema, progressing through phases of relapse and remission without typical histological signs of immune complex deposition [2]. A broader clinical definition emphasizes massive proteinuria and edema, with or without hypoalbuminemia [3]. The diagnosis is primarily clinical, with renal biopsy reserved for steroid-resistant cases [4]. Most children respond favorably to steroids, with complete remission of proteinuria in nearly 85% of patients after daily oral prednisone [5]. Steroid-sensitive nephrotic syndrome (SSNS) achieves remission within 4–6 weeks, whereas steroid-resistant nephrotic syndrome (SRNS) does not [6].

Evidence supports an immunological basis for INS, involving dysregulation of T helper 1 (Th1) and T helper 2 (Th2) cytokine balance [2]. The association of atopy with minimal change nephrotic syndrome (MCNS) suggests overlapping immune mechanisms, and several studies implicate Th2 cytokines in pathogenesis [5–8]. Cytokines are key mediators of inflammation and disease progression in INS [9–11]. The interleukin-1 (IL-1) family includes both proinflammatory (e.g., IL-1, IL-18, and IL-33) and anti-inflammatory (e.g., IL-36Ra, IL-37, and IL-38) members [12]. IL-38, a recently characterized cytokine produced in lymphoid tissues and by monocytes/macrophages, is implicated in infectious and autoimmune diseases [13]. The IL38 gene on chromosome 2q14.1 contains single nucleotide polymorphisms (SNPs) linked to susceptibility to various inflammatory and autoimmune conditions [14, 15].

INS typically develops in otherwise healthy children, with onset and relapses often associated with infections or immune triggers such as vaccinations or atopy [16–19]. Upper respiratory tract infections are common triggers [20–24], and COVID-19 has also been reported to precipitate relapse [25].

This study aimed to investigate the associations between serum IL-38 levels, the IL38 gene polymorphism rs7599662, and susceptibility to and severity of idiopathic nephrotic syndrome (INS) in pediatric patients. We aimed to ascertain whether IL-38 levels and rs7599662 genotypes could function as prospective diagnostic biomarkers and indicators of disease severity in INS.

## Results

### Baseline characteristics

The mean age of the NS patients was 7.9 ± 2.6 years, whereas that of the controls was 7.4 ± 2.6 years (*p* = 0.92). Males represented 72.7% of the cases and 66.2% of the controls (*p* = 0.39). The cohort included 50% steroid-sensitive, 25% steroid dependent, and 25% steroid-resistant patients. The demographic similarity between cases and controls reinforces the validity of subsequent biomarker and genetic analyses.

### rs7599662 genotype distribution

Genotype analysis revealed a significantly greater frequency of the CT genotype in cases than in controls (*p* = 0.003). A protective association was observed under the recessive model (TT vs. CT + CC, *p* = 0.002). The odds ratio (OR) for the TT genotype was 0.12 (95% CI: 0.03–0.55), indicating an approximately 88% reduced odds of INS among TT carriers. No significant associations were found with sex or clinical subtype (Table 1).

**Table 1:** Distribution of the rs7599662 genotypes in the study groups

Genotype/Allele	Cases (*N* = 88) No. (%)	Controls (*N* = 74) No. (%)	*P*-value
**Genotype**			
CC	2 (2.3%)	0 (0.0%)	0.003*
CT	84 (95.5%)	62 (83.8%)	
TT	2 (2.3%)	12 (16.2%)	
**Genetic Model**			
Dominant (CT + TT vs. CC)	86 (97.7%)	74 (100%)	0.50
Recessive (TT vs. CT + CC)	2 (2.3%)	12 (16.2%)	0.002*
**Allele Frequency**			
C allele	88 (50.0%)	62 (41.9%)	0.13
T allele	88 (50.0%)	86 (58.1%)	

*P-value calculated using Chi-square test; *P* < 0.05 considered statistically significant

### IL-38 Levels

Serum IL-38 levels were significantly lower in NS patients than in controls (17.9 ± 11.4 vs. 38.8 ± 35.6 ng/L, *p* < 0.001). The effect size of this difference, calculated as Cohen’s d, was 0.79, indicating a large and clinically meaningful reduction in circulating IL-38 in INS. No significant differences were observed by sex or among the clinical subtypes (Table 2).

**Table 2:** Serum IL-38 levels (ng/L) in the study groups

Groups	IL-38 (ng/L) Mean ± SD	Median (Range)	P-value
**Study groups (n=162)**			
Cases (n=88)	17.9±11.4	13.6 (5.2–59.8)	<0.001*
Controls (n=74)	38.8±35.6	21.3 (1.9–154.9)	
**Sex (n=162)**			
Males (n=113)	27.3±27.2	14.9 (1.92–154.9)	0.80
Females (n=49)	28.9±29.6	18.5 (6.6–111.8)	
**Subclinical cases type (n=88)**			
Steroid sensitive	18.1±13.02	12.6 (5.2–59.8)	0.32
Steroid dependent	16.8±8.7	13.1 (9.3–36.2)	
Steroid resistant	18.9±11.6	15.3 (7.6–49.9)	

*P < 0.05 considered statistically significant

### Clinical and laboratory findings by subtype

Compared with steroid-sensitive and steroid-dependent patients, steroid-resistant patients had significantly greater systolic and diastolic blood pressures, urine protein levels, albumin‒creatinine ratios (mean: 7.04 ± 5.6 mg/mg vs. 2.0 ± 3.1 mg/mg in SSNS, d = 1.10), urine protein (median: 2.4, IQR: 1.8–3.0 vs. 1.0, IQR: 0–2.0 in SSNS, *p* < 0.001), urine albumin levels, serum creatinine levels (0.76 ± 0.51 mg/dL vs. 0.43 ± 0.09 mg/dL in SSNS, d = 0.89), and urea levels (43.5 ± 34.3 mg/dL vs. 21.9 ± 5.4 mg/dL in SSNS, d = 0.88). (*p* < 0.05 for all). These patients also had higher urine pus and RBC counts (Table 3). The major differences in urinary protein, albumin-to-creatinine ratio, serum creatinine, and urea identified in the steroid-resistant cohort indicate the intrinsic disease severity of the SRNS phenotype, marked by pronounced glomerular injury and renal dysfunction (Table 3).

**Table 3:** Clinical and laboratory characteristics stratified by steroid response phenotype

Groups	Steroid sensitive (Mean ± SD)	Steroid dependent (Mean ± SD)	Steroid resistant (Mean ± SD)	*P*-value
**Clinical data**				
Age (years)	7.8±2.6	7.4±2.2	8.7±2.9	0.54
Weight (kg)	28.7±12.6	24.6±7.5	27.2±7.04	0.48
Systolic blood pressure	104.2±9.9	107.9±11.5	118.8±10.8	**0.05** ^**a**^ *, <0.001 ^**b**^ *, 0.005 ^**c**^ *
Diastolic blood pressure	57.4±7.5	65.1±8.2	72.7±10.3	**<0.001** ^**a**^ **'** ^**b**^ *, 0.01 ^**c**^ *
**Laboratory investigation**				
**Urine analysis**				
Pus	11.5±13.9	5.4±3.4	18.6±17.2	**0.04** ^**a**^ *, <0.001 ^**b**^ **'** ^**c**^ *
RBCS	10.2±20.9	2.8±1.1	10.9±8.01	**0.04** ^**a**^ *, <0.001 ^**b**^ **'** ^**c**^ *
PTN	1.04±1.2	1.27±1.2	2.4±0.90	0.37^a^,** <0.001**^b^*, 0.002^c^*
**Other labs**				
Albumin/Creatinine Ratio (mg/mg)	2±3.1	3.1±3.5	7.04±5.6	0.26^a^, **<0.001**^b^*, 0.01^c^*
Urine albumin (mg/dl)	195.1±447.8	169.3±368.8	516.4±474.6	0.93^a^, **<0.001**^**b**^**'**^c^*
Urine creatinine (mg/dl)	76.6±70.4	41.2±38.2	81.5±38.4	**0.02** ^**a**^ *, 0.006 ^**b**^ *, 0.002 ^**c**^ *
Serum creatinine (mg/dl)	0.43±0.09	0.41±0.12	0.76±0.51	0.78^a^, **0.03**^b^*, 0.02^c^*
Urea	21.9±5.4	22.4±4.5	43.5±34.3	0.68^a^,** 0.01**^b^*, 0.02^c^*

Data presented as mean ± SD. BP = blood pressure; ACR = albumin/creatinine ratio; HPF = high power field.^a^ Comparison between steroid-sensitive vs. steroid-dependent groups^b^ Comparison between steroid-sensitive vs. steroid-resistant groups^c^ Comparison between steroid-dependent vs. steroid-resistant groups*P < 0.05 considered statistically significant (Kruskal-Wallis test with post-hoc Dunn's test)

### Correlation analyses

**Fig. 1:**
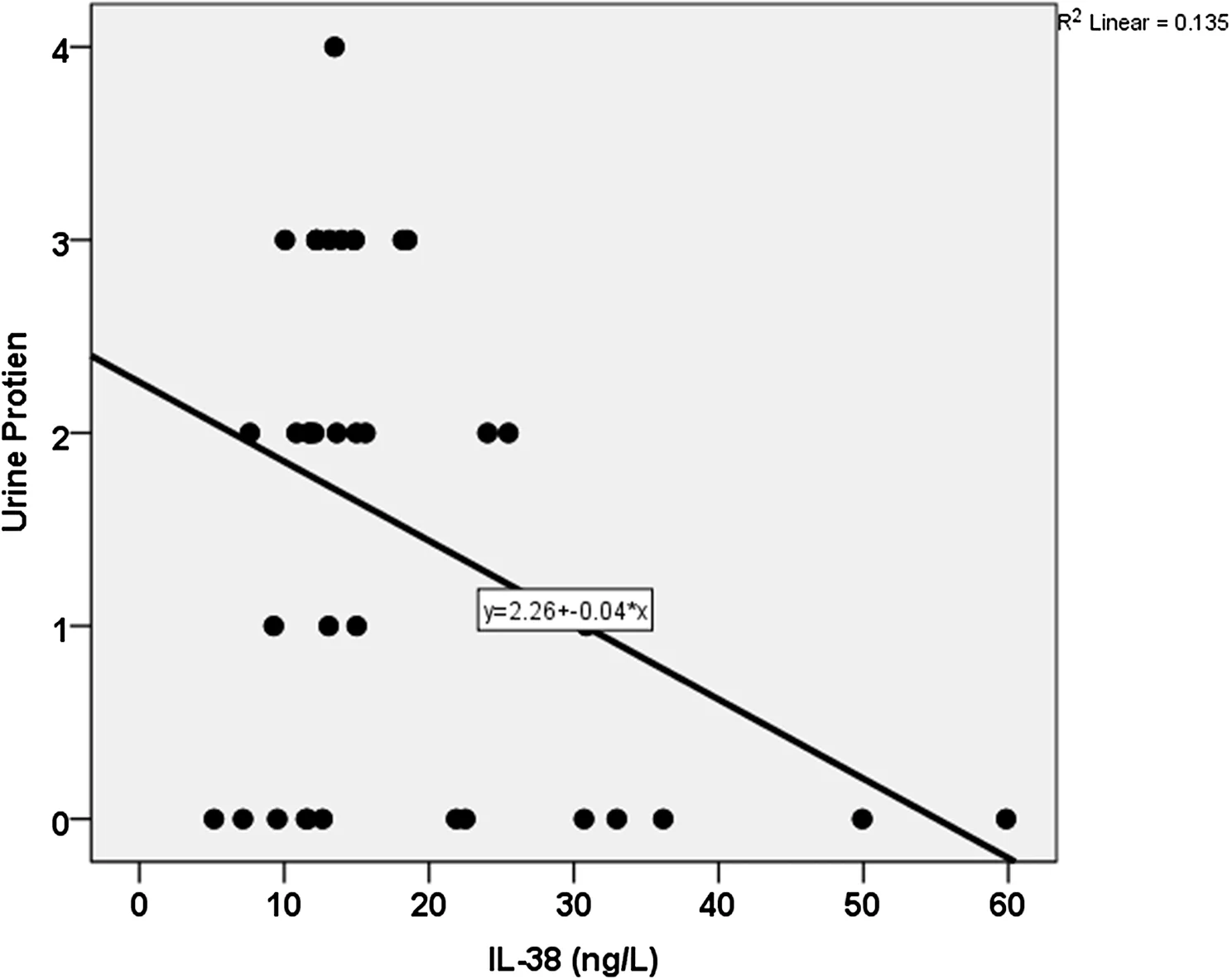
Scatter plot showing the significant negative correlation between serum IL-38 levels and urinary protein excretion (*r* = -0.38, *p* = 0.001)

IL-38 levels were significantly negatively correlated with patient age (*r* = -0.28, *p* = 0.01), systolic blood pressure (*r* = -0.28, *p* = 0.01), and urine protein (*r* = -0.38, *p* = 0.001). The IL-38 marker level showed a statistically significant negative correlation with urinary protein level with p-value 0.006 and < 0.001 respectively among steroid dependent, and resistant subgroups. However, there was no association between IL-38 and proteinuria level among steroid sensitive subgroup with p-value > 0.05 (Table 4; Fig. 1).

**Table 4:** Correlation analysis between serum IL-38 levels and clinical/laboratory parameters

Variables	IL-38 (ng/L) - r	*p*-value
**Clinical data**		
Age (years)	**−0.28**	0.01*
Weight (kg)	−0.20	0.08
Systolic blood pressure	**−0.28**	0.01*
Diastolic blood pressure	−0.12	0.29
**Laboratory investigations**		
**Urine analysis**		
Pus	−0.08	0.51
RBCS	0.08	0.52
PTN	**−0.38**	0.001*
**Other labs**		
Alb/Ctreat Ratio (mg/mg)	−0.22	0.06
Urine albumin (mg/dl)	−0.16	0.17
Urine creatinine (mg/dl)	−0.09	0.41
Serum creatinine (mg/dl)	−0.09	0.39
Urea	0.06	0.64
IL-38 vs. Proteinuria by Steroid-Response Subgroup		
Steroid sensitive	-0.24	0.18
Steroid dependent	-0.57	0.006*
Steroid resistant	-0.79	<0.001*

Spearman's correlation analysis performed. Correlation strength interpretation: |r| < 0.3 = weak; 0.3–0.7 = moderate; >0.7 = strong *P < 0.05 considered statistically significant

### Diagnostic performance of IL-38

**Fig. 2:**
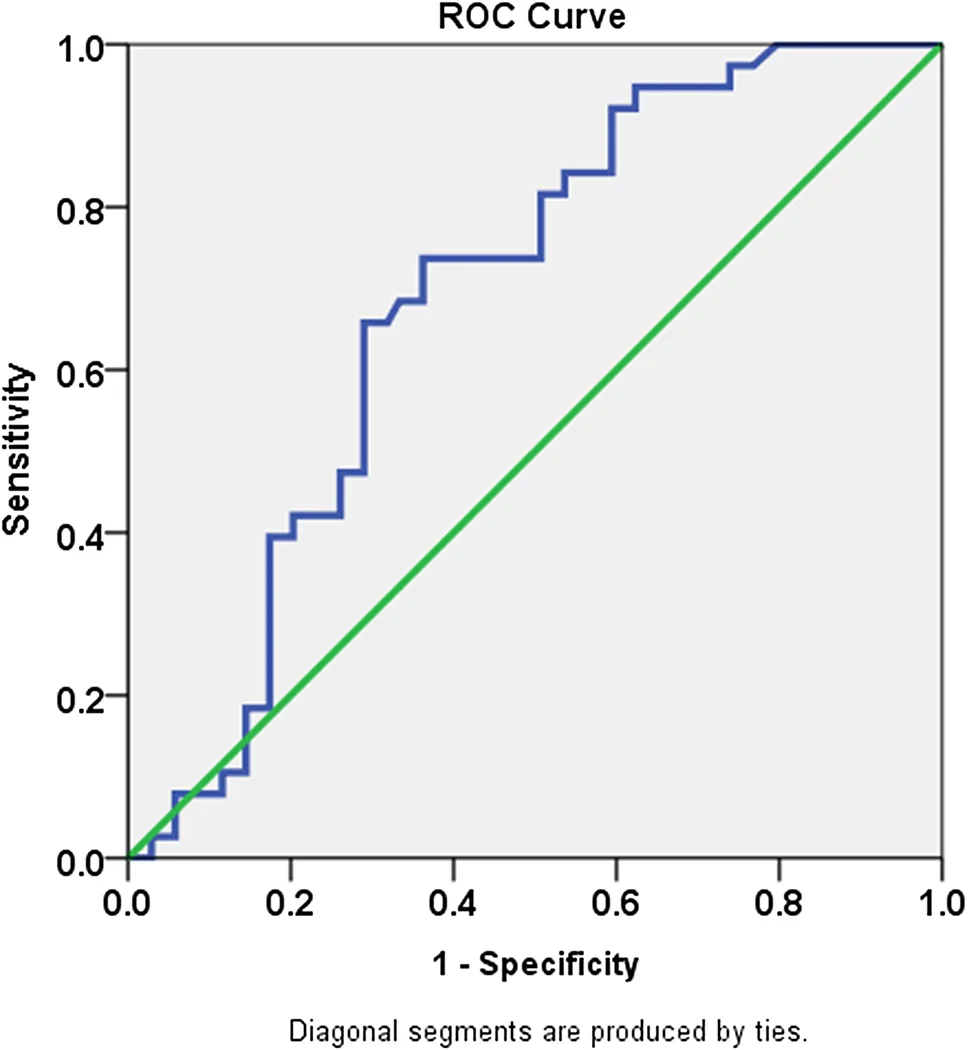
Receiver Operating Characteristic (ROC) curve for serum IL-38 in the diagnosis of nephrotic syndrome. The area under the curve (AUC) is 0.687 (95% CI: 0.598–0.777, *p* < 0.001). The diagonal reference line indicates no discriminatory power

ROC curve analysis indicated that IL-38 had moderate diagnostic performance for NS, with an AUC of 0.687 (95% CI: 0.598–0.777, *p* < 0.001). The area under the curve (AUC) was 0.687 (95% CI: 0.598–0.777, *p* < 0.001), indicating moderate diagnostic accuracy. At a cutoff of 18.773 ng/L, the sensitivity was 73.7%, and the specificity was 63.8%. At an optimal cutoff value of 18.773 ng/L, IL-38 demonstrated a sensitivity of 73.7% and a specificity of 63.8%. This suggests that nearly three-quarters of INS patients would be correctly identified by low IL-38 levels, though about one-third of healthy individuals may also fall below this threshold. The positive predictive value (PPV) and negative predictive value (NPV) in our cohort were 71.2% and 66.7%, respectively. These parameters support the potential of IL-38 as a complementary diagnostic biomarker, particularly in conjunction with standard clinical criteria (Fig. 2). These parameters indicate that IL-38 is probably not a good standalone diagnostic marker, but it could be useful as an extra part of a larger diagnostic or pathophysiological evaluation.

## Methods

### Study design and participants

This case‒control study enrolled 162 participants: 88 pediatric INS patients and 74 age- and sex-matched healthy controls. Patients were recruited from the pediatric department and outpatient clinic at Fayoum University Hospital between October 2024 and April 2025. Blood samples were obtained during active relapse, before initiation of the treatment episode. The controls were healthy children with no history of renal or systemic disease. Written informed consent was obtained from all parents or guardians. The study was approved by the Institutional Ethics Committee of Fayoum University Hospital (IRB number R659).

### Inclusion and exclusion criteria

The inclusion criterion was children aged 1–12 years with a diagnosis of nephrotic syndrome. The exclusion criteria were as follows: onset of NS before 1 year of age, active infection within 3 months prior to enrollment, estimated glomerular filtration rate (eGFR) < 60 mL/min/1.73 m², and secondary NS (e.g., Alport syndrome, lupus nephritis). Patients who had a renal biopsy as part of their regular clinical care (for example, because they were resistant to steroids or had an unusual presentation) were not excluded. However, histopathological data from biopsies were neither collected nor analyzed in accordance with this biomarker study protocol.

### Clinical definitions

Nephrotic syndrome (NS) is clinically defined by the presence of edema accompanied by significant proteinuria, which can be quantified as protein excretion exceeding 40 mg/m² per hour, a urine protein-to-creatinine ratio of ≥ 2000 mg/g (or ≥ 200 mg/mmol), or a dipstick reading of > 3 + proteinuria, in conjunction with serum albumin levels below 2.5 g/dL. The clinical course of INS is characterized by fluctuating phases of disease activity. Remission is achieved when proteinuria substantially resolves, as indicated by trace or negative urine albumin on dipstick, a protein excretion rate of less than 4 mg/m²/h, or a sustained urinary protein-to-creatinine ratio below 200 mg/g for three consecutive days. Conversely, a relapse is diagnosed upon the return of heavy proteinuria, marked by a urine albumin concentration of 3 + or 4 + on a dipstick, protein excretion exceeding 40 mg/m²/h, or a protein-to-creatinine ratio above 2000 mg/g for three consecutive days. Patients are classified as having Frequently Relapsing NS if they experience two or more relapses within any six-month period or four or more within a twelve-month period. Steroid-dependent NS is diagnosed when a patient experiences two consecutive relapses either during the tapering of corticosteroid therapy or within two weeks of its discontinuation. The most severe treatment response category is steroid-resistant NS, which is defined as failure to achieve remission after an intensive course of 4–8 weeks of daily oral prednisolone at a dosage of 2 mg/kg/day [1].

### Clinical and laboratory assessment

Demographic, anthropometric, and clinical data were recorded. Blood pressure was measured, and a comprehensive medical history was taken. All laboratory assessments were performed during active relapse, prior to the initiation of steroid therapy for the current disease episode. The laboratory parameters included urinalysis, the albumin-to-creatinine ratio, and the serum creatinine and urea levels. Histopathological data from renal biopsies were not included in this study’s protocol.

### Sample collection and analysis

Venous blood (5 mL) was collected under aseptic conditions. The serum used for IL-38 measurement was stored at -20 °C. Genomic DNA was extracted from EDTA blood for IL38 rs7599662 (C/T) genotyping via real-time PCR with a TaqMan SNP genotyping assay.

### Statistical analysis

The data were analyzed via SPSS v22. Descriptive statistics are presented as the means ± SDs, medians (ranges), or numbers (%). The Mann‒Whitney U test and the Kruskal‒Wallis test were used for quantitative data, and the chi‒square test was used for qualitative data. For significant Kruskal-Wallis results, post-hoc Dunn’s test with Bonferroni correction was applied. Spearman correlation and receiver operating characteristic (ROC) curve analyses were performed. A p value < 0.05 was considered significant.

### Sample size calculation

The sample size was calculated using G*Power software version 3.1.7. Based on preliminary data, a sample size of 22 patients per subgroup was determined to achieve a power of 85% with a two-sided type I error of 0.05 and an effect size of 0.753. The final cohort of 88 patients and 74 controls met this requirement.

### Reporting standard

This study was conducted and reported in accordance with the STROBE (Strengthening the Reporting of Observational Studies in Epidemiology) guidelines for case-control studies.

## Discussion

This study provides, to our knowledge, the first evidence of reduced serum IL-38 levels and an association of the IL38 rs7599662 polymorphism with pediatric INS. Our findings introduce a novel immunological dimension to the pathogenesis of this complex disorder and highlight a potential new player in the cytokine network that governs glomerular health and disease.

Nephrotic syndrome (NS) represents a complex clinical entity that is broadly classified as primary, originating from intrinsic kidney pathology, or secondary, resulting from systemic diseases. Primary NS itself can be subdivided into genetic and acquired (nongenetic) forms [26]. The cornerstone of initial management for idiopathic nephrotic syndrome (INS) is corticosteroid therapy. This approach creates a critical clinical divide: while steroid-sensitive nephrotic syndrome (SSNS) has an excellent prognosis with a minimal risk of progression to chronic kidney disease [1], 10–20% of patients with steroid-resistant nephrotic syndrome (SRNS) face dramatically different outcomes, with a 50% risk of end-stage renal disease within five years of diagnosis [27]. Our cohort reflected this well-established clinical heterogeneity, with cases distributed among steroid-sensitive, steroid dependent, and steroid-resistant phenotypes. A paramount challenge in managing INS is the unpredictable interindividual response to glucocorticoids. Although effective in inducing remission in 85–90% of juvenile cases by broadly suppressing proinflammatory cytokine synthesis [28], the current paradigm identifies SRNS only after a failed trial of therapy. This approach subjects nonresponders to unnecessary steroid toxicity and delays the initiation of alternative, potentially more effective, treatments. Therefore, the discovery of early predictive biomarkers for steroid resistance is a critical unmet need in nephrology. Our clinical data corroborate the severity of the SRNS phenotype, showing that steroid-resistant patients presented with significantly greater systolic and diastolic blood pressure and elevated markers of renal impairment than their steroid-sensitive counterparts did, findings likely compounded by prolonged, yet ineffective, glucocorticoid exposure.

The pursuit of such biomarkers has previously focused on proinflammatory mediators. For example, elevated levels of the chemokines CXCL8/IL-8 have been correlated with disease activity in NS. Research indicates that podocytes express receptors for CXCL8/IL-8 and can themselves release this chemokine, suggesting that high urinary levels signify significant podocyte injury and active inflammation [4, 29]. This persistent inflammatory state is thought to underpin the treatment resistance and frequent relapses observed in patients with SRNS and dependent SSNS. In this context, our study introduces interleukin-38 (IL-38) as a novel and significant player in INS. A key strength and novel finding of our work is that, to the best of our knowledge, this is the first study to investigate serum IL-38 levels in idiopathic nephrotic syndrome patients.

We observed a statistically significant reduction in serum IL-38 levels in patients with INS compared with controls, coupled with a significant negative correlation with urine protein levels. This finding is consistent with the established role of IL-38 as a potent anti-inflammatory cytokine. Reduced circulating IL-38 has been consistently reported in a spectrum of immune-mediated conditions, including Graves’ disease and Hashimoto’s thyroiditis [30], psoriasis vulgaris [31, 32], vitiligo [33], and gout [34]. Specifically, IL-38 has been shown to antagonize IL-36-driven inflammation in psoriatic skin, highlighting its relevance in Th17/IL-1 pathway dysregulation [31], which are pathways that are increasingly implicated in the pathogenesis of NS. The observed deficiency in IL-38 in our INS cohort suggests a failure of endogenous anti-inflammatory mechanisms, potentially creating a permissive environment for the unchecked inflammation that drives glomerular injury and proteinuria. We hypothesize that this IL-38 deficiency may disrupt the delicate balance between pro- and anti-inflammatory signals in the glomerular microenvironment, contributing to podocyte stress and breakdown of the filtration barrier.

The urine test markers for SRNS in our study were significantly elevated compared to those for SSNS and SDNS. The markedly increased indicators of proteinuria and renal dysfunction in steroid-resistant patients—comprising urinary protein, albumin-to-creatinine ratio, serum creatinine, and urea—are essential for the interpretation of our results. It is crucial to underscore that these differences signify intrinsic disease biology rather than treatment effects. All samples were obtained during active relapse prior to the introduction of steroid therapy for the current episode. By definition, SRNS is a more severe phenotype in which podocyte damage and glomerular filtration barrier dysfunction continue even after treatment. Consequently, the identified laboratory anomalies indicate the extent of underlying glomerular damage and advancing renal dysfunction associated with steroid resistance, rather than iatrogenic effects of treatment. This distinction emphasizes that the clinical and biochemical severity in SRNS represents a manifestation of the underlying disease pathophysiology.

Glomerular protein loss may elucidate the diminished serum IL-38 levels in INS. Urinary loss during active nephrosis may markedly reduce IL-38 levels owing to its low molecular weight (16.9 kDa) [35, 36]. Other cytokines, such as IL-6 and IL-8, have been reported to show similar behavior in proteinuric circumstances. Our results indicate a robust negative correlation between serum IL-38 and proteinuria in steroid-resistant patients, a modest correlation in dependent patients, and an absence of correlation in sensitive individuals, which is consistent with this notion. The elevated albumin-to-creatinine ratio in SRNS relative to SSNS and SDNS indicates that the strength of correlation intensifies with the severity of proteinuria. This indicates that renal excretion of IL-38 may reduce serum levels in severe proteinuric phenotypes. The absence of a notable correlation in steroid-sensitive individuals, characterized by reduced IL-38 levels, indicates that immune-mediated downregulation or compromised synthesis may have divergent effects, particularly in conditions with lesser proteinuria. Consequently, diminished IL-38 levels in INS are probably attributable to glomerular leakage, proteinuria, and a deficiency in anti-inflammatory signaling within the immunological milieu [36]. Additional research quantifying urine IL-38 and evaluating its fractional excretion is necessary to elucidate these contributions and the primary mechanism across clinical features.

A key finding of our study is that serum IL-38 levels did not differ significantly among the clinical subtypes of INS—steroid-sensitive, steroid-dependent, and steroid-resistant patients. This negative result is critical for interpreting the potential clinical role of IL-38. It suggests that while IL-38 is strongly associated with the presence of INS, it does not appear to be a useful biomarker for predicting or differentiating individual responses to glucocorticoid therapy.

It is essential to differentiate between the diagnostic and predictive functions of IL-38. Our observations indicate that IL-38 possesses moderate diagnostic efficacy in distinguishing INS patients from healthy controls. The absence of notable variations in IL-38 levels between steroid-responsive subgroups suggests that IL-38 does not serve as a predictive biomarker for treatment response. This differentiation is essential for understanding its therapeutic function. Serum IL-38 seems to indicate illness prevalence and the severity of proteinuria, rather than the specific immunological mechanisms that dictate steroid responsiveness.

Several interpretations of this finding are possible. First, it may reflect the limited statistical power of our subgroup analyses (*n* ≈ 22 per group), which were underpowered to detect subtle differences in IL-38 levels among phenotypes. Second, and more compellingly, it may indicate that IL-38 functions primarily as a marker of disease susceptibility and glomerular pathology rather than a modulator of the specific immune pathways that determine steroid responsiveness. This is supported by our correlation data, which show a strong negative association between serum IL-38 and proteinuria, particularly in steroid-resistant patients (*r* = -0.79). This gradient of correlation strength mirrors the severity of proteinuria across subgroups, implying that serum IL-38 levels are closely tied to the magnitude of glomerular barrier injury and consequent protein loss. The lack of correlation in steroid-sensitive patients suggests that in milder disease, IL-38 reduction may be less driven by urinary wasting and perhaps more related to baseline immunological dysregulation.

Therefore, we propose that in INS, serum IL-38 is best understood as an integrated biomarker reflecting the combined effects of glomerular protein leakage and underlying anti-inflammatory deficiency. Its reduction signifies a compromised anti-inflammatory state associated with disease presence and severity, but not necessarily with the specific therapeutic trajectory. This distinction is crucial for guiding future research: efforts should focus on validating IL-38 as a diagnostic and pathophysiological marker and on identifying other cytokine or molecular signatures that are specifically linked to steroid resistance mechanisms.

Furthermore, our study provides the first evidence of a genetic association between the IL38 rs7599662 polymorphism and nephrotic syndrome, another major novel contribution. Our analysis revealed a significant association under a recessive model, with a markedly lower frequency of the TT genotype in the patient cohort than in the control cohort. This finding suggests a potential protective effect of this genetic variant against INS susceptibility. A notable observation was the predominance of the CT genotype across all clinical subgroups, meriting further investigation in larger cohorts to determine whether it confers a generalized risk or influences disease presentation. This genetic finding is consistent with a previous report by Karaawi et al., who reported a similar protective effect of the rs7599662 TT genotype in COVID-19 patients [37]. To our knowledge, these two studies represent the entirety of the literature correlating this specific polymorphism with human disease, positioning our work as a pioneering genetic investigation in nephrology. The convergence of these findings across two distinct immune-related conditions strengthens the hypothesis that the IL38 locus plays a fundamental role in modulating host inflammatory responses.

Our study uncovers a significant genetic-phenotypic dissociation: the IL38 rs7599662 TT genotype exhibited a considerable protective association against INS in a recessive model, whereas serum IL-38 levels did not show significant variation among genotypes (Table 5). This apparent paradox, in which a genetic variant affects disease risk without changing the measurable levels of its encoded protein in the blood, necessitates meticulous mechanistic analysis. The most plausible hypothesis is that rs7599662 influences a “susceptibility threshold” for immune activation in response to environmental triggers, rather than constitutively altering steady-state IL-38 protein levels. The massive proteinuria in active nephrosis may obscure any subtle genotype-driven differences in IL-38 production through overwhelming urinary loss, thereby equalizing circulating levels across genotypes. Alternatively, the polymorphism may exert local functional effects within renal tissues or immune compartments that are not reflected in systemic blood measurements, or it may affect IL-38 protein function (e.g., receptor binding affinity or signaling potency) rather than its quantity. These hypotheses require direct testing in future functional studies.

**Table 5:** Association between rs7599662 genotypes and serum IL-38 levels

rs7599662 genotyping	IL-38 levels - Median	Range	P-value
**Dominant module**			
CT+ TT	15.6	1.92–154.9	0.14
CC	11.5	11.5–11.5	
**Recessive module**			
CT+CC	15.6	1.92–154.9	0.58
TT	17.5	6.6–21.9	
**Alleles**			
C allele	15.6	1.92–154.9	0.58
No C allele	17.5	6.6–21.9	
T allele	15.6	1.92–154.9	0.14
No T allele	11.5	11.5–11.5	

Mann-Whitney U test used for comparison. P < 0.05 considered statistically significant

However, the precise functional consequences of the rs7599662 polymorphism remain to be elucidated. It is plausible that this SNP affects IL38 gene expression or the stability of its mRNA, ultimately influencing the circulating and tissue-specific levels of the IL-38 protein. The lack of a significant difference in IL-38 levels among the clinical subtypes (SSNS, SDNS, and SRNS) in our study, despite the genetic association, suggests a complex relationship. It is possible that the polymorphism sets a susceptibility threshold, while the final disease phenotype and steroid response are determined by a combination of other genetic, epigenetic, and posttranslational factors. This underscores the need for integrated omics approaches in future research.

Given its moderate performance, IL-38 is unlikely to serve as a standalone diagnostic test. Future research should instead explore IL-38 as part of a multi-marker panel combining inflammatory, genetic, and renal injury biomarkers. Integration with established clinical parameters (e.g., proteinuria severity, serum albumin) and genetic risk scores may improve overall diagnostic and prognostic accuracy.

We acknowledge many study limitations that should be considered when interpreting our findings. First, we acknowledge the limitation of not quantifying urine IL-38 or fractional excretion. Without these data, we cannot determine whether the disease’s immunological dysregulation or glomerular protein loss through the compromised filtration barrier are the main causes of the lower serum IL-38 levels. Future prospective studies should use coupled blood and urine IL-38 measurements and fractional excretion analysis to determine the main mechanism across clinical characteristics.

Second, while all blood samples were taken before starting treatment for the current relapse episode, prior corticosteroids exposure in relapsing illness patients may be confusing. This past steroid use may have affected the immunological milieu and IL-38 expression without affecting disease activity. To better understand the function of IL-38 in disease etiology, future studies should start with treatment-naïve individuals or use a uniform corticosteroid washout period for those with prior exposure.

Third, it is important to note the constraints of our sample size for specific analyses. Our power calculation was designed to ensure robustness for the primary case-control comparison. Consequently, the study was not specifically powered to detect statistically significant differences among the treatment response subgroups (steroid-sensitive, dependent, and resistant). Therefore, the comparisons across these clinical phenotypes should be interpreted as exploratory and hypothesis-generating, underscoring the need for larger, specifically designed studies to investigate IL-38 in the context of treatment response.

A further significant limitation is the absence of histopathological correlation. Renal biopsy, the diagnostic gold standard in nephrology, is typically indicated in cases of steroid resistance or atypical presentation to define the underlying glomerular pathology, such as minimal change disease or focal segmental glomerulosclerosis [38]. The lack of biopsy data in our cohort prevents us from correlating serum IL-38 levels with specific histological diagnoses. Future research incorporating protocol biopsies or analyzing biomarkers within well-characterized histopathological cohorts will be essential to determine if IL-38’s role differs across distinct disease entities.

Finally, our work focused on IL-38 in the immunological axis of INS. We did not analyze metabolic factors like lipid profiles or immunological profiling, such as T-cell subsets (Th1, Th2, Th17, Treg), other important cytokines, or related IL-1 family members. The IL-38 deficit cannot be placed in a broader immunological or metabolic context or correlated with specific immune states like Th1/Th2 imbalance. Integrated immunophenotyping should be prioritized in future mechanistic research to define IL-38’s immunological circuits and its potential as a disease modulator beyond proteinuria.

## Conclusion

In conclusion, our research presents the initial evidence of diminished serum IL-38 levels and a correlation between the IL38 rs7599662 polymorphism and pediatric INS. The robust inverse correlation between serum IL-38 and proteinuria, especially in steroid-resistant instances, indicates that serum IL-38 probably signifies disease severity and glomerular protein loss rather than acting as a predictor of therapeutic response. Its failure to distinguish between clinical subtypes emphasizes its function as a diagnostic and pathophysiological marker rather than a biomarker of steroid responsiveness. The protective correlation of the TT genotype underscores a possible genetic factor influencing disease susceptibility. Serum IL-38 exhibits moderate diagnostic efficacy; however, its clinical relevance seems to be in detecting disease presence and indicating proteinuria burden. IL-38 did not exhibit predictive significance for treatment response in our cohort. Subsequent studies ought to integrate paired serum and urine IL-38 measurements within longitudinal cohorts to clarify the roles of urinary leakage versus immune regulation, and to discover more reliable biomarkers that accurately predict treatment outcomes. These results establish a basis for additional research on IL-38 as part of a multi-marker panel for INS and highlight the significance of integrated omics methodologies in comprehending this complex disorder. The hypothesis-generating nature of this work should be emphasized, and future confirmatory studies—including larger, adequately powered prospective cohorts—are required to validate these findings and establish clinical utility.

## Supplementary Information

Below is the link to the electronic supplementary material.


Supplementary Material 1



Supplementary Material 2


## Data Availability

The datasets generated and/or analyzed during the current study, and the materials used, are available from the corresponding author on reasonable request. No custom code or software was generated as part of this study.

## Abbreviations

AUC: Area Under the Curve
BMI: Body Mass Index
CI: Confidence Interval
eGFR: Estimated Glomerular Filtration Rate
ELISA: Enzyme-Linked Immunosorbent Assay
ESRD: End-Stage Renal Disease
HPF: High-Power Field
IL-38: Interleukin-38
INS: Idiopathic Nephrotic Syndrome
NS: Nephrotic Syndrome
PCR: Polymerase Chain Reaction
ROC: Receiver Operating Characteristic
SD: Standard Deviation
SNP: Single Nucleotide Polymorphism
SRNS: Steroid-Resistant Nephrotic Syndrome
SSNS: Steroid-Sensitive Nephrotic Syndrome
