# Supplementary material for: Serum IL-38 and the rs7599662 polymorphism: novel biomarkers for pediatric idiopathic nephrotic syndrome
Source: Ital J Pediatr. 2026 Aug 27;52:134. doi: 10.1186/s13052-026-02327-1 (PMC13523273; doi:10.1186/s13052-026-02327-1)
Supplement: Supplementary file 1 [file 13052_2026_2327_MOESM1_ESM.docx]

**Response to Reviewer Comments**

*Serum IL-38 and the rs7599662 Polymorphism: Novel Biomarkers for Pediatric Idiopathic Nephrotic Syndrome*

We thank the Reviewer for the careful evaluation of our manuscript and for the constructive comments. Below we provide a point-by-point response to each comment (Reviewer comments in italics, our responses in plain text). All changes have been made directly in the revised manuscript file.

# **Comment 1**

*“There are several white pages in the pdf submitted (and one also with an unclear subheading title.”*

**Response:** We thank the Reviewer for flagging this. On inspection, the submitted file retained several unused sections and blank formatting breaks carried over from the Springer Nature LaTeX/Word template on which the manuscript was originally built. Specifically, two placeholder sections — “Details on reference citations” and “Examples for theorem like environments” (both marked “Not Applicable”) — had not been deleted, and were separated from the surrounding text by several empty template paragraphs and an unnecessary landscape section break. Together these produced a run of blank and near-blank pages between the Results/Tables section and the Methods section, and “Details on reference citations” is very likely the unclear subheading the Reviewer noted, as it does not correspond to any content in our manuscript.

**Action taken:** We have removed both placeholder sections in their entirety, along with all associated empty paragraphs and the extraneous section break, so that the Results/Tables section now flows directly into the Methods section with no intervening blank pages. This is also consistent with the journal's formatting guidance to avoid manual page breaks in the manuscript. The revised file has been reduced.

# **Comment 2**

*“Overall the manuscript is not fully reported according to the journal's instructions.”*

**Response:** We have re-checked the manuscript against the Italian Journal of Pediatrics submission guidelines. In addition to removing the leftover template sections and page breaks described in our response to Comment 1, we confirmed that the manuscript uses double-line spacing with continuous line and page numbering throughout, and that the required Declarations subsections (Data Availability, Funding, Competing Interests, Author Contributions, Ethics Approval, Consent to Participate, Consent for Publication) are present and completed. We remain happy to make any further structural adjustments the Editor or Reviewer identifies as still being non-conforming.

# **Comment 3**

# *“Overall please temper the language in the whole manuscript (eg terms such as ‘we demonstrated’.. should be reworded given the study design and limited sample size).”*

**Response:** We agree that, given the case-control design and the sample size (particularly for the steroid-response subgroup comparisons, n ≈ 22 per group), several phrases in the Discussion overstated the strength of causal inference that the data support. We have revised the manuscript throughout to replace deterministic or causal language with more appropriately hedged, associative wording. Specific changes include:

| **Original text** | **Revised text** |
| --- | --- |
| “We demonstrated a statistically significant reduction in serum IL-38 levels...” | “We observed a statistically significant reduction in serum IL-38 levels...” |
| “This finding aligns perfectly with the established role of IL-38...” | “This finding is consistent with the established role of IL-38...” |
| “Other cytokines, such as IL-6 and IL-8, have demonstrated this in proteinuric circumstances.” | “Other cytokines, such as IL-6 and IL-8, have been reported to show similar behavior in proteinuric circumstances.” |
| “...thereby substantiating this notion.” | “...which is consistent with this notion.” |
| “...unequivocally demonstrates that IL-38 does not serve as a predictive biomarker for treatment response.” | “...suggests that IL-38 does not serve as a predictive biomarker for treatment response.” |

These revisions are confined to interpretive language in the Discussion; no changes were made to the reported results, statistics, or figures. The Conclusion section, which was already appropriately hedged (e.g., describing the findings as “hypothesis-generating” and calling for validation in larger prospective cohorts), is unchanged.

We thank the Reviewer again for their time and constructive feedback, which we believe has improved the clarity and rigor of the manuscript.
